# Serial ganglion impar blocks in a patient with nutcracker syndrome refractory to left renal vein transposition: a case report

**DOI:** 10.1186/s13256-020-02398-6

**Published:** 2020-07-03

**Authors:** Shawn Banon, Ioannis Skaribas

**Affiliations:** 1 Baylor College of Medicine, Houston, TX USA; 2 Expert Pain Care, Houston, TX USA

**Keywords:** Nutcracker syndrome, Left renal vein transposition, Ganglion impar block

## Abstract

**Background:**

Nutcracker syndrome is a rare disorder caused by compression of the left renal vein, usually between the aorta and the superior mesenteric artery. It typically presents with left-sided abdominal pain and hematuria. Left renal vein transposition is the most commonly employed surgical technique to alleviate the compression.

**Case presentation:**

A 22-year-old Caucasian man with a known diagnosis of nutcracker syndrome had undergone left renal vein transposition 1 year before presentation without any subsequent pain relief. In addition, his surgery was complicated by massive blood loss and a 1-week-long stay in an intensive care unit (ICU); as such, he was not amenable to further surgical intervention or stenting to treat his underlying pathology. His symptoms included constant sharp left-sided flank, perineal, and testicular pain. A series of ganglion impar blocks were performed every 3–4 months over the course of 5 years with substantial pain relief achieved.

**Conclusions:**

Our case report highlights a treatment option that has not yet been described for patients with pain secondary to nutcracker syndrome refractory to surgical intervention.

## Introduction

Nutcracker syndrome is a disorder caused by compression of the left renal vein. The vein is usually compressed between the aorta and the superior mesenteric artery (SMA), known as “anterior nutcracker”; an anatomic variant of the left renal vein may course between the aorta and vertebra, where it can also be compressed, known as “posterior nutcracker” [1]. Proposed causes of compression include posterior renal ptosis, abnormally high course of the left renal vein, abnormal branching of the SMA from the aorta, pancreatic neoplasm, para-aortic lymphadenopathy, retroperitoneal tumor, and abdominal aortic aneurysm [2–6]. The syndrome is most commonly associated with left-sided flank pain, hematuria (either frank or microscopic), orthostatic proteinuria, and orthostatic intolerance [7–9]. Microscopic hematuria is four times more common than frank hematuria [10]. Hematuria occurs due to rupture of veins in the renal fornix secondary to renal vein hypertension [11]. Renal vein hypertension also causes inflammation, which is thought to cause the flank pain; alternatively, blood clots can form in the collecting system, causing flank pain as well [12, 13]. The flank pain may radiate to the buttocks, posteromedial thigh, or groin [13, 14]. In addition, the pain can be exacerbated by sitting, standing, or walking [15]. Symptoms range from asymptomatic microhematuria to severe and persistent pain. In females, it can alternatively present as pelvic congestion, characterized by dysmenorrhea; dyspareunia; postcoital ache; lower abdominal pain; dysuria; fatigue; and pelvic, vulvar, or thigh varices [16, 17]. In addition, males can present with lower limb varices and varicoceles [18]. Autonomic dysfunction, including hypotension, syncope, and tachycardia, may be seen, although this is a rare presentation [19]. The prevalence of the syndrome is unknown, but it may have a female predominance [20]. Patients may present anywhere from childhood to the seventh decade of life, although most present in their second or third decade, with a possible second peak in middle-aged women [21]. Typically, Doppler ultrasound is performed as an initial assessment, and the diagnosis is confirmed with renal angiography, computed tomographic angiogram, digital subtraction angiography, magnetic resonance imaging (MRI), or magnetic resonance angiography [13].

**Fig. 1:**
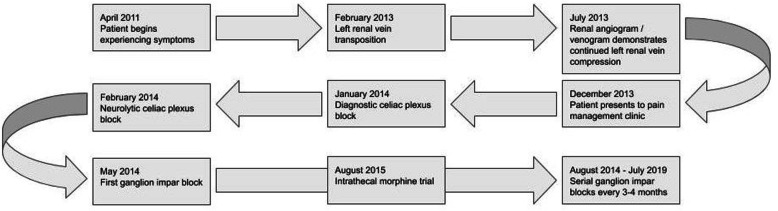
Chronology of treatment

## Case presentation

A 22-year-old Caucasian man with a medical history of epilepsy, nephrolithiasis, and nutcracker syndrome presented with abdominal and perineal pain. The patient’s family history was negative for nutcracker syndrome and pelvic congestion syndrome. The pain was ongoing for approximately 3 years before the initial consultation, and a left renal vein transposition was undertaken 1 year before initial consultation, without any improvement in his symptoms (Fig. 1). The pain was located in the left flank as well as variably in the left lower quadrant and left lower back. The pain consistently radiated to the groin and perineum and variably radiated to the left posterior thigh. The patient described the pain as sharp, shooting, and piercing. His pain was constant, with a baseline intensity of 6/10 on the visual analogue scale. His pain improved with medications, including gabapentin and hydrocodone-acetaminophen, restricting movement, and lying flat; it worsened with movement and sitting upright or standing. Associated signs included a varicocele, which had been repaired 6 years before initial consultation, and associated symptoms included infrequent nausea and vomiting. The patient’s vital signs were within normal limits. His physical examination revealed tenderness to palpation over the left flank without rebound tenderness or guarding. His laboratory test values were normal, except for microscopic hematuria on urinalysis. Due to continued flank pain at 6 months postoperatively, a repeat renal angiogram/venogram was obtained, which showed that although the left renal vein was successfully transposed, the left renal vein remained compressed with continued mild reflux into the left adrenal and left lumbar veins. Because our patient’s pain variably radiated into his posterior thigh, MRI of the lumbar spine was conducted, which showed mild bilateral L4 and L5 foraminal stenosis and mild disc bulge with mild mass effect on the left L5 nerve root. Even so, the results of straight leg raise and reverse straight leg raise tests were negative. In addition, the results of electromyography of the sciatic nerve and its branches were normal.

The patient underwent a diagnostic celiac plexus block with 70% improvement in his symptoms for 6 days, indicating a high likelihood of visceral origin of his pain. A celiac plexus neurolytic block was subsequently performed but relieved only 60% of his pain for 2 months. He was administered an injection of 5 ml of 0.25% bupivacaine followed by 15 ml of 98% dehydrated alcohol using the central technique. Although his flank pain was almost entirely relieved, his perineal and testicular pain, which was far more bothersome, persisted. As such, a ganglion impar block was undertaken, which significantly improved his pain (80% for 3 months). The block included 15 ml of 0.25% bupivacaine with 15 mg of dexamethasone. As a result, the patient underwent serial ganglion impar blocks over the course of 5 years, which significantly improved his symptoms (70–100%, lasting for 3–4 months). Some residual mild left flank pain persisted at times. Ganglion impar neurolysis was proposed; however, the patient declined for two reasons. Primarily, he was fearful of the possibility of perineal and testicular dysesthesias and hyperesthesias. Secondarily, because pain relief from neurolysis generally lasts no more than 6 months, he did not think the benefits outweighed the risks, considering that he was already benefiting from long-lasting pain relief from his current intervention. Although ganglion impar blocks were relieving his symptoms well, the patient wanted to explore more permanent options after approximately 2 years of consultation. For this reason, an intrathecal morphine trial was undertaken, but he was dissatisfied because of excessive pruritus and constipation and only a 60% reduction in pain. Later, a dorsal root ganglion trial was proposed; however, the patient had hesitation concerning permanent implantation of a device at that time. No adverse or unanticipated events have occurred as a result of his ganglion impar blocks.

## Discussion

Treatment of nutcracker syndrome varies, depending on the patient’s age and severity of symptoms. Pediatric patients have a higher rate of spontaneous remission, and, as such, patients younger than 18 are usually surveilled for 2 years versus 6 months in adults. Seventy-five percent of pediatric patients will have complete resolution of their hematuria in this time period [22]. This is thought to be secondary to an increase in intra-abdominal fibrous tissue at the origin of the SMA and an increase in retroperitoneal adipose tissue, which may increase the SMA–aorta angle and relieve tension on the left renal vein; the development of venous collaterals may play a role as well [12, 23, 24]. Surgeries are reserved for patients with severe and persistent symptoms in whom conservative treatment has failed. Surgical therapies include medial nephropexy, left renal vein bypass, left renal vein transposition, SMA transposition, renal autotransplant, gonadocaval bypass, renal to inferior vena cava inferior vena cava (IVC) shunt, and nephrectomy [1–3, 9, 15, 21, 24, 25]. In addition, the left renal vein can be stented endovascularly [26].

Left renal vein transposition is the most commonly employed surgical technique and is currently considered the gold standard on the basis of good outcomes and relatively low complication rates [23]. It involves excision of the vessel from the inferior vena cava (IVC), repair of the vena caval defect, and reanastomosis more distally along the inferior vena cava (IVC). In a 2002 study, seven of eight patients who received left renal vein transposition had resolution of their symptoms in from 41 to 136 months, with one patient experiencing persistent hematuria. Postoperative complications included deep vein thrombosis, retroperitoneal hematoma, and mechanical ileus [27]. Similarly, a 2009 study showed that nine of ten patients who underwent the surgery had improvement or resolution of their flank pain at 11 to 149 months, whereas hematuria resolved in all patients. Complications included chylous ascites, retroperitoneal hematoma, and left renal vein thrombosis [28]. Another 2009 study showed that six of seven patients who underwent the surgery had resolution of their symptoms in from 14 to 122 months, with one patient experiencing persistent pain; no postoperative complications were noted [29]. Our patient was in the minority of patients who did not experience pain relief from the surgery. In addition, because his surgery was complicated by massive blood loss and a 1-week-long stay in an intensive care unit (ICU), he was hesitant to undergo another surgery or stenting and opted to have his symptoms treated by a pain management physician instead.

The decision to undergo ganglion impar blocks rather than repeat celiac plexus neurolysis stemmed from our patient’s response to the interventions. The majority of his pain was in the testicular and perineal area, with only a minority in the left flank. As a result, our patient experienced more pain relief with ganglion impar blocks. However, the minority of pain not covered was usually in his left flank as a result. The patient was aware that risks of repeated steroid injections include weight gain, insulin resistance, and increased blood pressure; however, he felt that the benefits outweighed the risks. In addition, the patient was aware that his pathology persisted despite his pain relief. Overall, the patient has been satisfied with his results thus far.

## Conclusion

We report the successful treatment of abdominal, perineal, and testicular pain in a 22-year-old man with nutcracker syndrome. Our case report highlights a treatment option that has not yet been described for patients with pain secondary to nutcracker syndrome refractory to surgical intervention.

## Data Availability

Data sharing not applicable to this study, because no datasets were generated or analyzed during the current study.

## References

[1] Ali-El-Dein B, Osman Y, Shehab El-Din AB, El-Diasty T, Mansour O, Ghoneim MA, *et al.* Transplant Proc. 2003;35(2):851–3. 10.1016/s0041-1345(02)04026-5 12644163

[2] Wendel RG, Crawford ED, Hehman KN. The “nutcracker” phenomenon: an unusual cause for renal varicosities with hematuria. J Urol. 1980;123(5):761–3. 10.1016/s0022-5347(17)56121-2 7420571

[3] Hohenfellner M, Steinbach F, Schultz-Lampel D, *et al.* The nutcracker syndrome: new aspects of pathophysiology, diagnosis and treatment. J Urol. 1991;146(3):685–8. 10.1016/s0022-5347(17)37893-x 1875472

[4] Shokeir AA, el-Diasty TA, Ghoneim MA. The nutcracker syndrome: new methods of diagnosis and treatment. Br J Urol. 1994;74(2):139–43. 10.1111/j.1464-410x.1994.tb16574.x 7921927

[5] Skeik N, Gloviczki P, Macedo TA. Posterior nutcracker syndrome. Vasc Endovascular Surg. 2011;45(8):749–55. 10.1177/1538574411419376 21890560

[6] Menard MT. Nutcracker syndrome: when should it be treated and how? Perspect Vasc Surg Endovasc Ther. 2009;21(2):117–24. 10.1177/1531003509338402 19703821

[7] Buschi AJ, Harrison RB, Norman A, *et al.* Distended left renal vein: CT/sonographic normal variant. AJR Am J Roentgenol. 1980;135(2):339–42. 10.2214/ajr.135.2.339 6773339

[8] Ekim M, Bakkaloglu SA, Tümer N, Sanlidilek U, Salih M. Orthostatic proteinuria as a result of venous compression (nutcracker phenomenon)—a hypothesis testable with modern imaging techniques. Nephrol Dial Transplant. 1999;14(4):826–7. 10.1093/ndt/14.4.826 10328451

[9] Takahashi Y, Sano A, Matsuo M. An ultrasonographic classification for diverse clinical symptoms of pediatric nutcracker phenomenon. Clin Nephrol. 2005;64(1):47–54. 10.5414/cnp64047 16047645

[10] Shin JI, Park JM, Lee JS, Kim MJ. Effect of renal Doppler ultrasound on the detection of nutcracker syndrome in children with hematuria. Eur J Pediatr. 2007;166(5):399–404. 10.1007/s00431-006-0252-7 PMC1829442 17051355

[11] Pournasiri Z. The nutcracker syndrome as a rare cause of chronic abdominal pain: a case report. J Compr Pediatr. 2016;7(3):e39741.

[12] He Y, Wu Z, Chen S, *et al.* Nutcracker syndrome—how well do we know it? Urology. 2014;83(1):12–7. 10.1016/j.urology.2013.08.033 24139744

[13] Kurklinsky AK, Rooke TW. Nutcracker phenomenon and nutcracker syndrome. Mayo Clin Proc. 2010;85(6):552–9. 10.4065/mcp.2009.0586 PMC2878259 20511485

[14] Baril DT, Polanco P, Makaroun MS, Chaer RA. Endovascular management of recurrent stenosis following left renal vein transposition for the treatment of Nutcracker syndrome. J Vasc Surg. 2011;53(4):1100–3. 10.1016/j.jvs.2010.10.112 21215570

[15] Coolsaet BL. Ureteric pathology in relation to right and left gonadal veins. Urology. 1978;12(1):40–9. 10.1016/0090-4295(78)90365-5 98890

[16] d’Archambeau O, Maes M, De Schepper AM. The pelvic congestion syndrome: role of the “nutcracker phenomenon” and results of endovascular treatment. JBR-BTR. 2004;87(1):1–8. 15055326

[17] Zhang H, Li M, Jin W, San P, Xu P, Pan S. The left renal entrapment syndrome: diagnosis and treatment. Ann Vasc Surg. 2007;21(2):198–203. 10.1016/j.avsg.2006.10.021 17349362

[18] Little AF, Lavoipierre AM. Unusual clinical manifestations of the nutcracker syndrome. Australas Radiol. 2002;46(2):197–200. 10.1046/j.1440-1673.2001.01037.x 12060163

[19] Daily R, Matteo J, Loper T, Northup M. Nutcracker syndrome: symptoms of syncope and hypotension improved following endovascular stenting. Vascular. 2012;20(6):337–41. 10.1258/vasc.2011.cr0320 22734085

[20] Shin JI, Lee JS, Kim MJ. The prevalence, physical characteristics and diagnosis of nutcracker syndrome. Eur J Vasc Endovasc Surg. 2006;32(3):335–6. 10.1016/j.ejvs.2006.04.030 16781173

[21] Rudloff U, Holmes RJ, Prem JT, Faust GR, Moldwin R, Siegel D. Mesoaortic compression of the left renal vein (nutcracker syndrome): case reports and review of the literature. Ann Vasc Surg. 2006;20(1):120–9. 10.1007/s10016-005-5016-8 16374539

[22] Tanaka H, Waga S. Spontaneous remission of persistent severe hematuria in an adolescent with nutcracker syndrome: seven years’ observation. Clin Exp Nephrol. 2004;8(1):68–70. 10.1007/s10157-003-0267-6 15067520

[23] Ananthan K, Onida S, Davies AH. Nutcracker syndrome: an update on current diagnostic criteria and management guidelines. Eur J Vasc Endovasc Surg. 2017;53(6):886–94. 10.1016/j.ejvs.2017.02.015 28356209

[24] Scultetus AH, Villavicencio JL, Gillespie DL. The nutcracker syndrome: its role in the pelvic venous disorders. J Vasc Surg. 2001;34(5):812–9. 10.1067/mva.2001.118802 11700480

[25] Shaper KR, Jackson JE, Williams G. The nutcracker syndrome: an uncommon cause of haematuria. Br J Urol. 1994;74(2):144–6. 10.1111/j.1464-410x.1994.tb16575.x 7921928

[26] Chiesa R, Anzuini A, Marone EM, *et al.* Endovascular stenting for the nutcracker phenomenon. J Endovasc Ther. 2001;8(6):652–5. 10.1177/152660280100800620 11797984

[27] Hohenfellner M, D’Elia G, Hampel C, Dahms S, Thüroff JW. Transposition of the left renal vein for treatment of the nutcracker phenomenon: long-term follow-up. Urology. 2002;59(3):354–7. 10.1016/s0090-4295(01)01537-0 11880069

[28] Reed NR, Kalra M, Bower TC, Vrtiska TJ, Ricotta JJ 2nd, Gloviczki P. Left renal vein transposition for nutcracker syndrome. J Vasc Surg. 2009;49(2):386–94. 10.1016/j.jvs.2008.09.051 19216958

[29] Wang L, Yi L, Yang L, *et al.* Diagnosis and surgical treatment of nutcracker syndrome: a single-center experience. Urology. 2009;73(4):871–6. 10.1016/j.urology.2008.11.043 19193424

